# Characterization of microRNA expression profiles in normal and osteoarthritic human chondrocytes

**DOI:** 10.1186/1471-2474-13-144

**Published:** 2012-08-12

**Authors:** Silvia Díaz-Prado, Claudia Cicione, Emma Muiños-López, Tamara Hermida-Gómez, Natividad Oreiro, Carlos Fernández-López, Francisco J Blanco

**Affiliations:** 1 Department of Medicine, INIBIC-University of A Coruña, A Coruña, Spain; 2 CIBER-BBN-Cellular Therapy Area, INIBIC-Hospital Universitario A Coruña, A Coruña, Spain; 3 Rheumatology Division, INIBIC-Hospital Universitario A Coruña, A Coruña, Spain; 4 Tissue Bank, INIBIC-Hospital Universitario A Coruña, A Coruña, Spain; 5 Osteoarticular and Aging Research Laboratory. Biomedical Research Center, Instituto de InvestigaciÃ³n BiomÃ©dica de A CoruÃ±a (INIBIC), Hospital Materno Infantil Teresa Herrera, C/As Xubias S/N. 15.006, A Coruña, Spain

**Keywords:** microRNA, Microarray, Chondrocyte, Osteoarthritis, Human

## Abstract

**Background:**

Osteoarthritis (OA) is a multifactorial disease characterized by destruction of the articular cartilage due to environmental, mechanical and genetic components. The genetics of OA is complex and is not completely understood. Recent works have demonstrated the importance of microRNAs (miRNAs) in cartilage function. MiRNAs are a class of small noncoding RNAs that regulate gene expression and are involved in different cellular process: apoptosis, proliferation, development, glucose and lipid metabolism. The aim of this study was to identify and characterize the expression profile of miRNAs in normal and OA chondrocytes and to determine their role in the OA.

**Methods:**

Chondrocytes were moved to aggregate culture and evaluated using histological and qPCR techniques. miRNAs were isolated and analyzed using the Agilent Human miRNA Microarray.

**Results:**

Of the 723 miRNAs analyzed, 7 miRNAs showed a statistically significant differential expression. Amongst these 7 human miRNAs, 1 was up-regulated in OA chondrocytes (hsa-miR-483-5p) and 6 were up-regulated in normal chondrocytes (hsa-miR-149*, hsa-miR-582-3p, hsa-miR-1227, hsa-miR-634, hsa-miR-576-5p and hsa-miR-641). These profiling results were validated by the detection of some selected miRNAs by qPCR. *In silico* analyses predicted that key molecular pathways potentially altered by the miRNAs differentially expressed in normal and OA chondrocytes include TGF-beta, Wnt, Erb and mTOR signalling; all of them implicated in the development, maintenance and destruction of articular cartilage.

**Conclusions:**

We have identified 7 miRNAs differentially expressed in OA and normal chondrocytes. Our potential miRNA target predictions and the signalling cascades altered by the differentially expressed miRNAs supports the potential involvement of the detected miRNAs in OA pathology. Due to the importance of miRNA in mediating the translation of target mRNA into protein, the identification of these miRNAs differentially expressed in normal and OA chondrocyte micropellets could have important diagnostic and therapeutic potential. Further studies are needed to know the function of these miRNAs, including the search of their target mRNA genes, which could lead to the development of novel therapeutic strategies for the OA treatment.

## Background

Osteoarthritis (OA) is a degenerative joint disease characterized by deterioration in the integrity of hyaline cartilage and subchondral bone. OA is the most prevalent articular pathology and the most frequent cause of disability. The etiology for OA is unknown but multiple factors such as obesity, age, anatomic abnormalities, history of joint trauma, joint instability, repeated injury, overuse and joint dysplasia are thought to be involved, resulting in severe joint pain, loss of movement, and irreversible functional disability with a marked decrease in quality of life [1,2]. This degenerative process is driven by the activation of the single cell type present in the mature cartilage, chondrocytes [3]. The incidence of OA is directly related to age and is expected to increase along with the median age of the population.

MicroRNAs (miRNAs) are single-stranded and small noncoding RNA molecules of 18–24 nt in length that negatively regulate the expression of target genes in a post-transcriptional manner. They bind to messenger RNAs, through incomplete base pairing with the 30-untranslated region (30-UTR) of targets mRNA, to suppress its gene expression via degradation or translational repression [4,5]. Depending on the degree of base pairing between the target mRNAs and the miRNA, the miRNA either cleavage the mRNAs (perfect pairing) or repress translation (imperfect pairing). Although some algorithms are used to predict potential mRNA targets, only a few miRNAs have been validated and assigned to specific mRNAs. These small RNAs are naturally produced by the cells and they are derived from primary miRNA transcripts (70 to 100 nt) that are processed in the nucleus to precursor miRNAs (pre-miRNAs) by the ribonuclease Drosha. Later, these pre-miRNAs are transported into the cytoplasm where they are further processed into miRNAs by the ribonuclease Dicer. A single miRNA can regulate the expression of many target genes, and a target gene can also be regulated by several miRNAs [6]. These short molecules are conserved from worms to mammals; this high conservation of miRNAs sequences highlights the significance of their function. They contribute to the regulation of a variety of biological functions across diverse organisms such as apoptosis, proliferation, differentiation, development, cell cycle, stem cell maintenance, metabolism and hematopoiesis [6-9]. Recently, specific miRNAs were reported to be involved in chondrogenesis and inflammatory cartilage diseases [10-15]. A recent work by Kobayashi et al. [16] demonstrated the role of miRNAs in cartilage function. These authors showed that Dicer, a critical enzyme for biogenesis of miRNAs, is essential for normal skeletal development; since they generated cartilage-specific Dicer-null mice that showed a greatly decreased chondrocyte proliferation and accelerated hypertrophy leading to severe growth defects and premature death of mice [16].

Recent evidences have also indicated that these small RNA molecules play a role in the pathogenesis of human disorders such as birth defects and cancer [5], exhibiting tissue-specific or developmental stage-specific expression patterns associated with human diseases. Whilst some studies suggested that miRNA genes account for more than 1,000 [6], predictive algorithms indicated that more than a third of all human genes contain putative single or multiple miRNA recognition elements [9]. MicroRNAs are encoded all over the genome, in intergenic regions, introns, exons, exon overlaps, and UTR regions, and approximately 50 % of known human miRNAs are found in clusters [17]. The clustered miRNAs are often related to each other, in terms of targeting the same gene or different genes in the same biochemical pathway, but can also be unrelated.

The study of miRNA has become a new field in life science. The detection of miRNA expression is a very important first step in miRNA exploration [6]. Current methodologies have been developed and applied successfully in miRNA profiling, including microRNA arrays [6]. The use of microarrays for global characterization of miRNA expression is becoming an increasingly popular research tool [18,19]. The growing interest in miRNAs has sparked a natural extension of microarray technology to screen the expression level of miRNA in parallel [4]. A number of human diseases are associated with changes in the copy number or expression of microRNAs, indicating that miRNA expression levels are closely associated with developmental and physiological states as well as disease process [6,8]. Several reports on miRNA profiling human cartilage [15], cancer [20] and general human tissues [19] have already been published. The expression profiles of miRNAs are effective for classification of human cancer [20], also several published studies showed a probable link between miRNAs and other human diseases [21], since microRNA signatures have been associated with well-defined clinicopathological features and disease outcome [22]. The potential utility of miRNA expression profiles in diagnosis and disease monitoring has been investigated and some studies have postulated the diagnostic and prognostic utility of circulating RNAs [23]. MiRNA gene signatures may be more useful and provide more discrimination than mRNA expression signatures [4]. In some cases it has been possible to successfully classify poorly differentiated tumors using the expression profiles of these microARNs while the mRNA profiles, from the same samples, were not adequate for this type of classification. Thus it appears that the expression profiles of miRNA are better than those of mRNA to classify tumors [20].

However, at present, little is known if aberrant microRNA expression is associated with OA development. For example, miR-146a, miR-155, miR-132 and miR-16 were found to be up-regulated in rheumathoid arthritis (RA) patients compared with OA ones [16,24,25]. Moreover, it was demonstrated that high levels of miR-146 and miR-16 expression were correlated with active disease, whereas low expression levels correlate with inactive disease [7]. Furthermore two recent works identified, by microarray and Real Time PCR assays, 16 and 17 microRNAs differentially expressed in OA compared to normal cartilage [9,15]. The problem is that both studies tested the expression of a small number of miRNAs, in particular 365 and 157 miRNAs respectively, of the total 1,048 different mature microRNAs identified in humans and recently released by the Sanger miRBase (release 16.0, September 2010) [26], although many more miRNAs are still to be identified. Also, contradictory results were postulated in these two recent studies. In this sense, Jones et al. [9] demonstrated that miR-25 is upregulated in OA chondrocytes whereas Iliopoulos et al. [15] stated that this miRNA was downregulated in OA cartilage. In the present study, to better understand the molecular mechanisms involved in the pathogenesis of OA, we identify and characterize the expression profiles of 723 human miRNAs from normal and OA chondrocytes, which could have important diagnostic and therapeutic potential.

## Methods

### Harvest of human cartilage and isolation of chondrocytes

Human cartilage samples, four healthy donors (3 males and 1 female with a mean age of 68 years and a range from 58 to 79 years) with a Mankin score of 1, and six III and IV grade OA donors (4 males and 2 females with a mean age of 64 years and a range from 42 to 80 years) with a Mankin score of 10 (additional file 1), were provided by the Autopsy Service and the Orthopaedic Department at Hospital Universitario A Coruña, Spain. These samples came from patient who underwent replacement surgery or limb amputations. This study was approved by the Ethic Committee of Clinical Investigation of Galicia, and informed consent was obtained from all donors.

Cartilage sections were aseptically removed from each donor, sliced full thickness and washed in Dulbecco′s modified Eagle′s medium (*DMEM*) (Gibco, Life Technologies, Paisley, Scotland) supplemented only with antibiotic penicillin (100 units/ml)-streptomycin (100 μg/ml) (Gibco Invitrogen, USA) as previously described [27]. Briefly, slices were minced with a scalpel and transferred to a digestion buffer containing 1% trypsin (Sigma, St. Louis, MO) for 15 min at 37°C until digestion was complete. The supernatant (without chondrocytes) was discarded and, after trypsin removal, the trypsinized cartilage was incubated in a second digestion buffer containing 2 mg/l of type IV Collagenase (Sigma, St. Louis, MO) for 12 to 16 h at 37°C overnight. After this time cells were washed three times with DMEM and centrifuged at 200 xg for 10 minutes before being used for culture. The number of chondrocytes obtained was counted by a Neubauer Chamber using the 0.4% tripan blue dye (Sigma, St. Louis, MO) to assess the viability of the sample.

### Chondrocyte culture

Chondrocytes were cultured in a 25 cm^2^ culture flask with DMEM supplemented with 100 units/ml penicillin, 100 μg/ml streptomycin, 1% glutamine and 10% FBS (Fetal Bovine Serum) (Gibco, Life Technologies, Paisley, Scotland) in humidified 5% CO_2_ atmosphere at 37°C (Steri-Cult 200 Incubator HEPA class 100, Hucoa Erlöss). Chondrocytes in first sub-culture (S1) were employed for micropellet studies.

### Micropellet formation

Adherent cells in culture from different donors were treated with trypsin-EDTA. 5x10^5^ cells were centrifuged at 200x*g* for 10 minutes and the cellular aggregate was cultured in DMEM with 10% FBS for 1 week. The culture medium was changed every 3–4 days. 5 micropellets were developed for each of the donors. After 1 week the micropellets were quickly frozen or embedded in paraffin or included in OCT freezing medium and subsequently they were used for RNA isolation or for histological and immunohistochemical stainings.

### Histological and immunohistochemical analyses

For general histological analyses, 4 μm-thick paraffin sections of micropellets were deparaffinized in xylol, rehydrated in a graded series of ethanol, and stained with Hematoxylin-Eosin (HE), Alcian Blue (AB), Safranin O (SO) and Masson′s Trichromic (MT). HE staining allowed performing a general assessment of the structure of the micropellets, differentiating the nucleus of the cells with respect to their cytoplasms and the synthesised extracellular matrix. AB and SO stainings revealed the presence of proteoglycans. MT staining allowed performing a general assessment of the structure of the micropellets, as in the HE staining, but also revealing the presence of collagens.

Frozen sections (4 μm-thick) were incubated with different primary antibodies to detect the presence of collagen types I (Abcam, Spain) and II (BioNova Científica, Spain), aggrecan C-20 (Santa Cruz Biotechnology Inc., Germany) and metalloproteinase 13 (Thermo Fisher Scientific, Spain). The peroxidase/DAB ChemMate^TM^ DAKO EnVisionTM detection kit (Dako Citomation, USA) was used to determine antigen-antibody interaction. Negative staining controls were achieved by omitting the primary monoclonal antibody. Samples were visualized using an optical microscope.

### RNA extraction

For aggrecan quantification we used qPCR analysis. Isolation of total RNA, coming from 2 to 3 micropellets from the same donor, was performed using Trizol Reagent (Invitrogen, Spain) according to manufacturer′s instructions. From each micropellet, 5x10^5^ cells were obtained for RNA isolation. Total RNA was further processed in RT-PCR or stored at −80°C until its use. RNA integrity was confirmed by 2% agarose gel electrophoresis and stained with ethidium bromide. RNA also was assessed for quantity at 260 nm using a NanoDrop^TM^ spectrophotometer (Thermo Scientific, Spain). A260/A280 relation was calculated for quality and purity.

For miRNA microarray and miRNA qPCR analyses, total RNA (including microRNAs) was isolated with *mirVana*^*TM*^*miRNA Isolation kit* (Applied Biosystems, Spain), according to manufacturer′s protocol, and analyzed by the DNA microarray hibridization Service at CNIO (*Centro Nacional de Investigaciones Oncológicas*, Spain). As a rigorous step, and before label reaction, samples were analyzed by means of a LabChip system using a 2100 Agilent Bioanalyzer (Agilent Technologies, Spain) in order to known RNA concentration and RNA Integrity Number (RIN). This analysis was designed to reveal the ability of RNA samples for the microarray hybridization experiment [28].

### miRNA microarray

Expression levels of 723 microRNAs (miRNA Sanger Base release 10.1) were studied using Human miRNA microarray kit (version 2) (Agilent Technologies, Spain). Total RNA fraction was used to determine its RIN, which were in the range of 7.4 to 9.6, by Lab-chip technology on an Agilent 2100 Bioanalyzer (Agilent Technologies, Spain). 120 ng of total RNA was labelled and hybridized using the commercial *miRNA Microarray System with miRNA Complete and Hyb Kit* (Agilent Technologies, Spain) by following manufacturer's instructions. The entire labelled sample was used for the hybridization reaction which was performed at 55 °C during 40 hours in a total volumen of 45 μl. Images were scanned on a G2565CA microarray scanner (Agilent Technologies, Spain) and quantified using Agilent Feature Extraction (FE) Software (ver. 10.1.1) (Agilent Technologies, Spain). Also, microarray data were normalized and analyzed using Agilent FeatureExtraction (FE) Software and GeneSpring GX10 (Agilent Technologies, Spain). All microarray hybridization experiments and data analysis were performed by the miRNA expression profiling Service of CNIO (Madrid, Spain). The sequence of events involved in the processing of the text raw data files is thresholding to 1 and normalization [=log (base 2) transformation and 75 percentile shift]. Baseline transformation has not been performed.

### cDNA synthesis

For aggrecan quantification reverse-transcription (RT-PCR) was performed from 1 μg of total RNA using SuperScript^TM^ First-Strand Synthesis System for RT-PCR (Invitrogen^TM^, Spain) up to a total volume of 20 μl in a Thermocycler (Gene Amp PCR System 9700, Applied Biosystem, Spain). 1 μg of total RNA, 2.5 nM random hexamers, 0.5 mM of dNTP mix, and 3 μl of DEPC-treated water were denatured at 65°C for 5 minutes and chilled on ice for at least 1 minute. On the other hand, 2 μl of 10xRT buffer, 5 mM MgCl_2_, 0.01 M DTT, and 40 U of RNaseOUT Recombinant Ribonuclease Inhibitor were mixed, collected by centrifugation, and incubated at 25°C for 2 minutes. After incubation, 50 U of SuperScript^TM^ RT were added and incubated at 25°C for 10 minutes, 42°C for 50 minutes and 70°C for 15 minutes in a Thermocycler (Gene Amp PCR System 9700, Applied Biosystem, Spain). Finally, samples were chilled on ice and incubated with 2 U of RNAse H for 20 minutes at 37°C before proceeding to amplification the target cDNA.

Samples were stored at −20 °C before cDNA target was amplified. Positive and negative controls were included in each experiment. RNA extraction, reverse transcription-PCR assay setup and post reverse transcription-PCR product analysis were carried out in separate designated rooms to prevent cross-contamination.

### Real-Time Quantitative PCR

PCR amplification of aggrecan mRNA was carried out using primers and conditions shown in Table 1, on LightCycler® 480 Instrument (Roche, Mannheim, Germany) using LightCycler 480 SYBR Green I Master (Roche, Mannheim, Germany).

**Table 1:** Primer sequences, conditions and annealing temperature of PCR assay for aggrecan mRNA amplification

**Primers**	**Sequence**	**Length**	**%GC**	**Annealing Temperature**	**Amplicon (bp)**
AGG 1 F	5′ GCCTACGAAGCAGGCTATGA 3′	20 mer	55	60°C	136
AGG 1R	5′ GCACGCCATAGGTCCTGA 3′	18 mer	61		
HPRT 1 F	5′ TGACCTTGATTTATTTTGCATACC 3′	24 mer	33	60°C	102
HPRT 1R	5′ CGAGCAAGACGTTCAGTCCT 3′	20 mer	55		

An initial activation at 95°C for 5 minutes was followed by an amplification target sequence 50 cycles of 95°C for 10 s, 60°C for 10s, and 72°C 7 s were used. For melting curve analysis 1 cycle of 95°C for 5 s, 70°C for 15 s, and 95°C for 1 s was used. Finally, a cooling step was used at 40°C for 10 s.

We verified that amplifications and the expected size of each PCR product were specific. 1.8% agarose gel electrophoresis of all PCR products revealed a single band that corresponded to the single-amplified products as predicted by the melting curve analysis of the PCR. Each assay was done at least in triplicate and included marker-positive and marker-negative controls and reagent with no template controls.

PCR primers for mRNA amplification were carefully designed using the web-based ProbeFinder software (Universal ProbeLibrary Design Center) accessible via Roche Applied Science home page [29]. PCR primers have been positioned to span exon-intron boundaries, reducing the risk of detecting genomic DNA. Primers were purchased from Roche (Mannheim, Germany).

PCR amplification of microRNAs (hsa-miR-145, hsa-miR-149, hsa-miR-483-5p, hsa-miR-582-3p, hsa-miR-634 and hsa-miR-641; as annotated in the miRBase ver 10.0 and 11.0) was carried out on the LightCycler® 480 Instrument (Roche, Mannheim, Germany) using miRCURY LNA^TM^ microRNA PCR System (Exiqon, Vedbaek, Denmark). U6 snRNA was used as endogenous control.

An initial activation at 95°C for 10 minutes was followed by an amplification target sequence 60 cycles of 95°C for 10 s and 60°C for 10s were used. For melting curve analysis 1 cycle of 95°C for 5 s, 60°C for 1 min, and 97°C for 1 s was used. Finally, a cooling step was used at 50°C for 20 s. Relative levels of expression were calculated by the 2^-ΔΔCt^[30].

### DNA Sequencing Analysis

After aggrecan quantification by qPCR, at least one PCR product coming from each PCR experiment was used as template DNA in order to verify that amplifications corresponded to the aggrecan cDNA. PCR products were purified by enzymatic method (ExoSAP-IT, Amersham Biosciences, Spain). DNA sequencing was performed in a reference facility on ABI 3100 (Applied Biosystems, Spain) using Big Dye terminators. Forward and Reverse specific primers were used.

### Other procedures

Standard procedures for manipulation of nucleic acids were essentially those of Sambrook et al. [31].

### Statistical analysis

All statistical analyses regarding miRNA microarray data were facilitated, together with the results, by the miRNA expression profiling Service of CNIO (Madrid, Spain). The miRNA data were analyzed with the GeneSpring GX10.0 software. In order to analyze the miRNAs with differential expression in healthy and OA donors, *T* test unpaired was used. For the cluster tree analysis, the k-means clustering algorithm was performed on all the samples. The rest of the analyses were performed using SPSS 17.0 software for Windows, p-values <0.05 were considered to be statistically significant.

### Bioinformatic analyses for mRNA target and molecular pathway prediction

Putative target genes regulated by the miRNAs differentially expressed in normal and OA chondrocyte micropellets were predicted bioinformatically and combining the prediction of their supposed targets with the bibliographic information of chondrocyte gene expression. For this purpose miRanda algorithm and the miRGen database were used. miRanda computes optimal sequence complementarity between a set of mature microRNAs and a given mRNA using a weighted dynamic programming algorithm [8]. miRGen is a database that aims to provide comprehensive information about the position of human and mouse microRNA coding transcripts and their regulation by transcription factors, including a unique compilation of both predicted and experimentally supported data [32]. miRanda and miRGen are freely available at [33] and [34]. Moreover the TargetScan Human resource, which provide miRNA target predictions based on sequence complementary to target sites with emphasis on perfect base-pairing in the seed region and sequence conservation [35], was used. TargetScan is freely available at [36]. These web-based computational tools are based on different algorithms which are based on several parameters calculated individually for each miRNA.

In order to identify molecular pathways potentially altered by the expression of multiple miRNAs, we used the DIANA-mirPath web-based computational tool [37], free available at [38]. The software performs an enrichment analysis of multiple miRNA target genes comparing each set of miRNA targets to all known KEGG (***K****yoto****E****ncyclopedia of****G****enes and****G****enomes*) pathways [39]. The combinatorial effect of co-expressed miRNAs in the modulation of a given pathway is taken into account by the simultaneous analysis of multiple miRNAs. The graphical output of the program provides an overview of the parts of the pathway modulated by microRNAs, facilitating the interpretation and presentation of the analysis results.

## Results

### Evaluation of chondrocyte micropellets of donors using histological, immunohistochemical and molecular biology techniques

**Figure 1:**
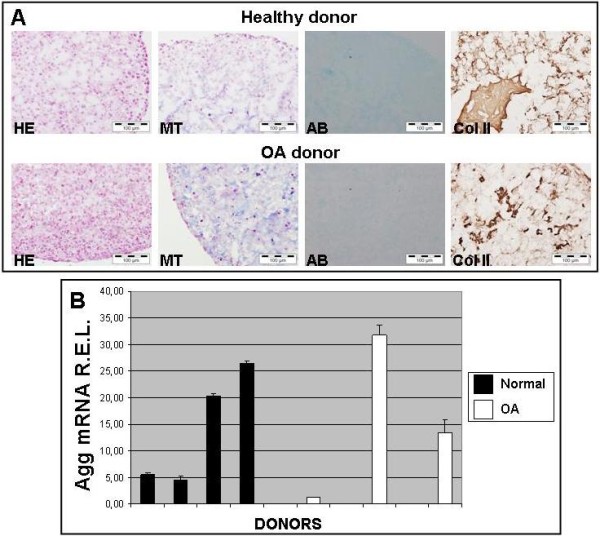
**Evaluation of chondrocyte micropellets of donors using histological, immunohistochemical and molecular biology techniques.** [**A**] Chondrocyte micropellets from normal and OA donors grown for 7 days in Dulbecco's Modified Eagle Medium (DMEM) supplemented with 10% FBS. Micropellets were stained with Hematoxylin-Eosin (HE), Alcian Blue (AB) and Masson′s Trichromic (MT). Immunodetection of type II collagen (Col II) was performed to detect this molecule which is characteristic of hyaline cartilage. [**B**] Aggrecan mRNA R.E.L. of healthy and OA chondrocyte micropellets measured by Real-Time Quantitative PCR (mean ± S.E.).

MicroRNA for the microarray studies were generated by extracting total RNA from chondrocyte micropellets of healthy (n = 4) and OA (n = 6) donors. These micropellets were analyzed, prior to RNA isolation, using different stainings in order to identify the specific and main components of the cartilage extracellular matrix. Specifically, we sought to determine the presence of molecules characteristics of hyaline cartilage, such as proteoglycans and collagens in general, and type II collagen in particular. As it is shown in Figure 1, chondrocyte micropellets from healthy and OA donors showed the typical structure of a micromass. In each micropellet two areas were observed; the peripheral zone that was very cellular and with low extracellular matrix, and the central area that had a greater amount of extracellular matrix synthesized by the cells.

Chondrocyte micropellets from healthy samples showed the presence of collagens, in general, and type II collagen in particular. Moreover, they were negative for MMP13 and type I collagen immunostainings (data not shown). Regarding Safranin O stainings, surprisingly all healthy chondrocyte micropellets from healthy donors showed absence or weakly presence of proteoglycans by histochemistry. Due to the limitations of this histological technique to detect low amounts of a specific compound, we therefore assessed the presence of aggrecan mRNA by qPCR. All healthy chondrocyte micropellets from healthy donors showed amplification of aggrecan mRNA. In this regard aggrecan mRNA R.E.L. ranged from 4.64 to 26.37 (mean ± S.E.: 14.22 ± 5.41).

On the other hand, chondrocyte micropellets of OA donors were also positive for alcian blue, Masson′s trichromic and type II collagen stainings whereas they were negative for MMP13 and type I collagen immunostainings (data not shown). As for the case of healthy donors, chondrocyte micropellets from OA patients showed absence or weakly expression of proteoglycans by histochemistry but by means of qPCR we detected the presence of aggrecan mRNA in 3 of 6 donors. For OA chondrocyte micropellets, aggrecan mRNA R.E.L. ranged from 0 to 31.8 (mean ± S.E.: 7.74 ± 5.27).

### MicroRNA profiling of normal and OA chondrocytes

**Figure 2:**
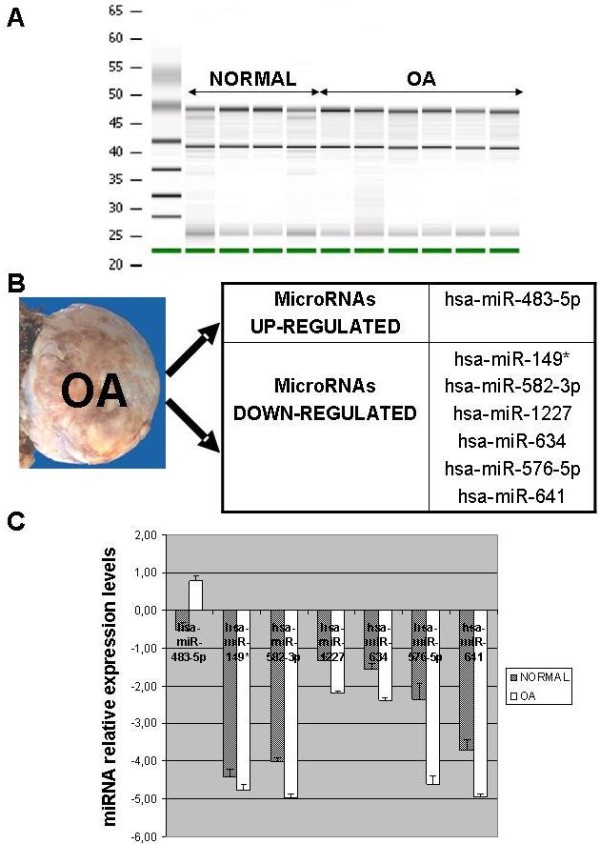
**BioAnalyser gel profiles of total RNA isolated from chondrocyte micropellets of healthy and OA donors were used to determine the RIN, RNA concentration and rRNA ratio [A].** Up-regulated microRNAs in OA and normal chondrocyte micropellets [**B**]. Relative expression levels of those microRNAs differentially expressed in normal and OA chondrocyte micropellets (p value < 0.1 and a fold-change cut-off >1.5) by Human miRNA microarray assay (mean ± S.E.) [**C**].

**Figure 3:**
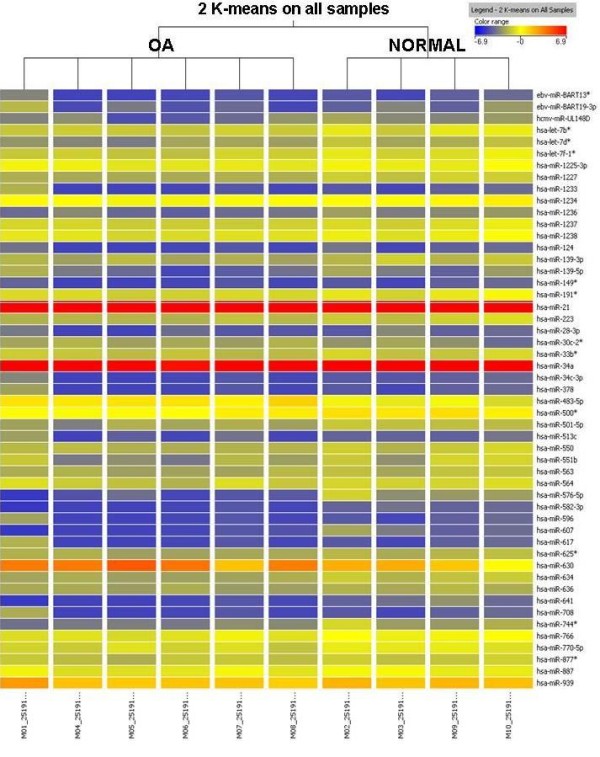
**Cluster tree showing miRNA gene expression data of OA and normal chondrocytes micropellets.***T* test unpaired (2 K-means on all samples) with no multiple testing correction was performed.

To assess the putative role of miRNAs in OA pathology, we performed a microarray analysis of six OA chondrocyte micropellets along with four normal chondrocytes micropellets. In order to obtain sufficient quantity of miRNA for subsequent microarray analysis it was necessary to extract total RNA from chondrocyte micropellets of healthy and OA donors. Total RNA fraction was used to determine the RNA Integrity Number (RIN), which was in the range of 7.4 to 9.6, and to assess RNA concentration and rRNA ratio [28 s/18 s] by means of a bioanalyzer (Figure 2A). Once samples passed this quality control they were ready to be labelled and hybridized with the Agilent Human miRNA Microarray version 2. This microarray allowed us to test the expression of 723 microRNAs in chondrocyte micropellets of healthy and OA donors. After raw data were processed and normalized, the microRNA profiling of normal and OA chondrocytes revealed a few number of miRNAs differentially expressed in normal and OA chondrocytes. Of the 723 miRNAs immobilized on the microarray only 7 miRNAs, with a fold-change cut-off >1.5, showed a statistically significant differential expression (Table 2). Amongst these 7 human miRNAs, 1 was up-regulated in OA chondrocytes (hsa-miR-483-5p) and 6 were up-regulated in normal chondrocytes in comparison to OA chondrocytes (hsa-miR-149*, hsa-miR-582-3p, hsa-miR-1227, hsa-miR-634, hsa-miR-576-5p and hsa-miR-641) (Figure 2 B and C). In this regard, hsa-miR-576-5p was down-regulated in OA chondrocyte pellets with the highest fold (4.74) whereas hsa-miR-483-5p was up-regulated in OA chondrocyte pellets with 2.44 fold. As it is shown in Figure 3, cluster tree contains results of K-means clustering algorithm performed with GeneSpring GX on all the samples. This cluster analysis was performed by K-means method, ie, defining that the clustering should identify two classes (normal and OA; K-means = 2). The algorithm separated into two main branches or groups, in this sense it separated in one cluster all OA chondrocyte micropellet samples and in the other all normal chondrocyte micropellet samples. This fast and efficient clustering technique allowed us to analyze miRNA gene expression data where the most similar expression profiles were joined together to form a group. In this regard, the miRNA expression profiles of all the samples analyzed allowed us to distinguish two clusters: OA and normal chondrocytes micropellets. Therefore, these 48 miRNAs could represent valid markers in discriminating normal versus OA chondrocyte samples, although the small numbers of samples analyzed on the miRNA microarray requires further studies.

**Table 2:** miRNA differentially expressed in normal (N) versus OA

**miRNA name**	**Sanger Accession**	**Fold Change**	**p value**
hsa-miR-483-5p	MIMAT0004761	2.44	2.35×10^-4^
hsa-miR-149*	MIMAT0000450	−1.76	2.28×10^-3^
hsa-miR-582-3p	MIMAT0004797	−1.94	3.31×10^-4^
hsa-miR-1227	MIMAT0005580	−1.80	2.54×10^-5^
hsa-miR-634	MIMAT0003304	−1.75	7.66×10^-4^
hsa-miR-576-5p	MIMAT0003241	−4.74	6.68×10^-4^
hsa-miR-641	MIMAT0003311	−2.36	1.71×10^-3^

Data from microarray analysis of miR expression was used to identify miRNAs with differential expression in normal versus OA micropellets. Only miRNAs with a statistically significant (p value < 0.1) differential expression and a fold-change cut-off >1.5 are listed. U Mann–Whitney test without Benjamin-Hochberg multiple testing corrections were used to compute p values. Fold change gives the absolute ratio of normalized intensities (no log scale) between the average intensities of the samples grouped (N versus OA). Positive fold change values indicate elevated expression in OA micropellets, whilst negative fold change values indicate elevated expression in normal micropellets.

### Real Time Quantitative PCR analyses of miRNAs differentially expressed in normal and OA chondrocytes micropellets

**Figure 4:**
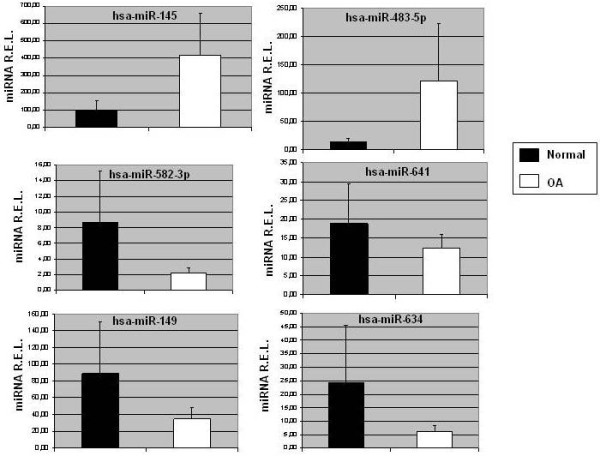
**Quantitative reverse transcription-polymerase chain reaction (RT-PCR) analysis was performed to quantify hsa-miR-145 and microRNAs differentially expressed in normal and OA chondrocyte micropellets (mean ± S.E.).** For this analysis 4 healthy and 6 OA samples were used.

We selected the hsa-miR-149, hsa-miR-483-5p, hsa-miR-582-3p, hsa-miR-634 and hsa-miR-641 differentially expressed for further quantification using quantitative PCR techniques (Figure 4). Because hsa-miR-145 showed elevated expression in OA chondrocytes, although it was not statistically significant, and it was previously published in the literature to be upregulated, together with hsa-miR-483, in osteochondromas compared to normal cartilage [40], we decided to select it for qPCR verification. The same comparison conditions were used for the qPCR analyses as for the microarray experiments. We compared miRNA expression in normal against OA chondrocyte micropellets. The total RNA isolated from the same normal and chondrocyte samples were used for qPCR. In this regard, hsa-miR-145 and hsa-miR-483-5p were also up-regulated in OA chondrocyte micropellets, in particular 4.4 and 8.45 fold respectively, according with the results obtained in the miRNA microarray analysis. On the other hand, hsa-miR-582-3p, hsa-miR-641, hsa-miR-149 and hsa-miR634 were down-regulated in OA chondrocyte micropellets, 3.9, 1.52, 2.6 and 4.03 fold respectively, in agreement with miRNA microarray data, although there were no statistical significant differences when comparing the different miRNA R.E.L. in normal versus OA chondrocyte micropellets (p > 0.05, *U* Mann–Whitney test). Therefore, the analysis of selected miRNAs by qPCR confirmed the microarray results, indicating the quality of the miRNA microarray. 

### Bioinformatic prediction of putative target mRNA genes regulated by the miRNAs differentially expressed in normal and OA chondrocytes micropellets

To pursue the study, we performed a bioinformatic prediction in order to know the putative target genes regulated by all the miRNAs differentially expressed in normal and OA chondrocyte micropellets. For this purpose the following computational tools were used: miRanda [33], miRGen [34] and TargetScan [36], which utilize distinct parameters to predict the probability of a specific miRNA to bind within the 3′-UTR sequence of a given mRNA gene. All the computational programs predicted potential target mRNA genes for the 7 miRNAs differentially expressed in normal and OA chondrocytes micropellets and also for the hsa-miR-145. These potential mRNA targets were grouped by their function. Of the 7 miRNAs differentially expressed, and the hsa-miR-145, the largest number of predicted putative targets included binding proteins (21 to 25%) except for the hsa-miR-483-5p whose largest number of putative targets included enzymes (18%). The number of transcription proteins obtained as putative mRNA targets regulated by hsa-miR-145, hsa-miR-576-5p and hsa-miR-1227 were also high (18% to 19%), whereas secretory, membrane, surface or receptor proteins as predicted targets regulated by hsa-miR-149, hsa-miR-483-5p, hsa-miR-582-3p, hsa-miR-634 and hsa-miR-641 were also elevated (15 to 19%). Lower percentages of potential targets were related with cell adhesion, translation, transporter proteins among others.

**Figure 5:**
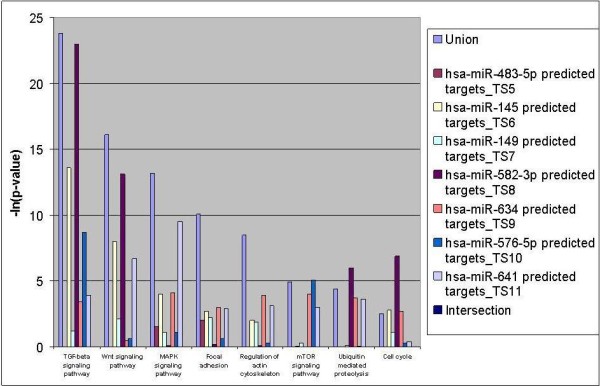
** DIANA-miRPath *in silico *analysis showing the genes targeted by the hsa-miR-145 and selected miRNAs grouped by molecular pathway.**

In order to obtain a list of pathways likely to be specifically controlled by the miRNAs differentially expressed in normal and OA chondrocytes micropellets, we performed an *in silico* analysis of the putative interactions between the 7 miRNAs (hsa-miR-1227 was not incorporated in the analysis because it was not included in the miRNA input list) and the most common signalling pathways by using the web-based computational tool named DIANA-miRPath. This computational tool estimates the impact of co-expressed miRNAs in biological pathways. Figure 5 represents the DIANA-miRPath analysis based on TargetScan 5 target prediction software. It shows the number of genes targeted by each miRNA. The “union” bar in a specific pathway indicates the coordinated downregulation of the pathway by all co-expressed miRNAs whereas the “intersection” bar reflects and overview of the cooperative downregulation of single genes by all of the expressed miRNAs. As it is shown in the graph, genes potentially interacting with these miRNAs were found to be involved in TGF-beta, Wnt, MAPK and mTOR signalling, focal adhesion and regulation of actin cytoskeleton among others.

## Discussion

OA is the most common disease of joints in adults around the world [41], being the knee OA the most common type. About one-third of all adults have radiological signs of OA. The likelihood of developing OA increases with age. The prevalence of OA of the knee is higher among 70- to 74-year-old. Epidemiological studies have revealed that there are both endogenous and exogenous risk factors for OA. The pathogenesis of OA is characterized by the progressive destruction of articular cartilage. Many factors contribute to the overall degradation of cartilage observed in OA, either directly or indirectly by modulating anabolic factors [14]. The capacity of articular cartilage to repair is very limited, mainly because it is an avascular tissue [42]. Currently, there are no effective pharmacological treatments to treat OA although some drugs slow its progression. Surgical treatments are not a way to treat OA even so they still constitute an important tool in cartilage repair of those injuries which if not treated or repaired properly could inevitably end up producing a secondary OA.

Some miRNA-related studies on cancers and genetic diseases are based on their different expression profile in pathologic compared to normal tissues [19,20]. Most of these studies postulate that differential expression of candidate miRNAs is a possible approach to investigate the function of miRNAs in human diseases. In attempting to understand the biological pathways and processes that underlie the pathogenesis and progression of OA, genomic approaches have identified miRNAs genes associated with cartilage development and homeostasis, extracellular matrix components and IL-1 signal transduction pathway among others [11,14,43,44]. Tissue miRNAs have been noted not only as key molecules in intracellular regulatory networks for gene expression, but also as biomarkers for various pathological conditions [45]. For example, the expression of miR-146 has been shown to be intensely expressed in low grade OA cartilage, indicating that it might play a role in OA cartilage pathogenesis [12]. In addition, it has been demonstrated that miR-27b regulates the expression of MMP13 (Matrix Metalloproteinase 13) in human OA chondrocytes, suggesting that up-regulation of miR-27b in vivo would represent a novel therapeutic approach in OA [44]. In recent years, a putative role for miR-140 in the pathogenesis of OA was evidenced [11,14,43], since its expression is significantly reduced in OA tissue and that in vitro treatment of chondrocytes with IL-1ß, a cytokine involved in the pathogenesis of OA, suppresses miR-140 expression [46]. Recent studies have also demonstrated that several miRNAs might play a role in OA pathogenesis [9,47].

In the present study, to better understand the molecular mechanisms involved in the pathogenesis of OA and to investigate a possible role of miRNAs in cartilage-related genes regulation and OA development, we comprehensively isolated and analyzed miRNAs of normal and OA chondrocyte micropellets, using miRNA microarray analysis. From a technical point of view, it is difficult to obtain a high number of chondrocytes from articular cartilage explants. Chondrocytes from a healthy cartilage represent only the 2% of the total volume of the cartilage. Therefore it is also complicated obtain large amounts of RNA, enriched in miRNAs, of excellent quality to performed the microarrays. This difficulty is even higher when using OA cartilage samples, since OA cartilage has a smaller number of cells than the healthy one. The micropellet model is different to the tissue, since it allows maintaining the cells in a three-dimensional position, these cells can synthesize extracellular matrix and allows obtaining a greater number of chondrocytes. For this reason chondrocytes micropellets, and not the cartilage explants, were employed for miRNAs isolation.

This study identified and characterized the expression profiles of 723 human miRNAs from normal and OA chondrocytes, of which 1 miRNA up-regulated in OA chondrocyte and 6 were up-regulated in normal chondrocyte micropellets. Unsupervised clustering performed by using processed data from miRNA microarray analysis highlighted differential expression profiles of 48 miRNAs, interestingly clustering the samples into 2 groups, OA versus normal chondrocyte micropellets. Therefore these miRNAs could represent valid markers in discriminating normal versus OA chondrocyte samples although further studies focused in a large number of samples should be performed to determine the potential of these miRNAs for clinical application in the diagnosis of OA pathology.

Our profiling results were further validated by the detection of some selected miRNAs by qPCR. Some of these selected miRNAs (e.g. hsa-miR-145 and hsa-miR-483-5p) have already been described in the literature [15,40,48]. Of particular interest was the finding that hsa-miR-483-5p was up-regulated in OA chondrocyte micropellets as previously described Iliopoulus et al., [15]. In this regard, Iliopoulos et al. [15] reported their finding of a 16-miRNA OA gene signature from their studies comparing osteoarthritic and nondiseased human cartilage. These authors found that hsa-miR-483-5p was upregulated in OA cartilage, not only by miRNA microarray analysis but also by qPCR techniques. These findings are in agreement with our miRNA microarray and qPCR results since we observed an upregulation of hsa-miR-483-5p in OA chondrocyte micropellets with the highest fold (8.45) obtained by qPCR. On the other hand, Zuntini et al. [40] also verified that hsa-miR-145 and hsa-miR-483 are both upregulated in osteochondromas when they are compared to normal cartilage. A recent study postulated that aberrant expression miR-483-5p together with miR-195 allow the identification of a subset of poorer prognosis adrenocortical carcinomas [49]. Moreover, Patterson et al. [50] found that the high expression of miR-483-5p appears to be a defining characteristic of adrenocortical malignancies, indicating that it can thus be used to accurately distinguish between benign and malignant adrenocortical tumors. However Dunn et al. [48], profiling miRNA expression in bovine articular cartilage, found that hsa-miR-145 were downregulated in monolayers of tissue cultured chondrocytes as compared with levels determined directly from intact native cartilage. Our microarray analyses showed that the relative expression levels for hsa-miR-145 were −2.87 for healthy and 1.85 for OA samples. Moreover, qPCR experiments showed that this miRNA was also up regulated in OA donors, in particular 4.4 fold. However neither the microarray nor the qPCR results achieve the statistical significance previously published in the literature. Perhaps it could be due to the use of different microarray technologies, or to the use of cultured cell instead of tissue samples.

In our study miR-149* was down regulated in OA chondrocyte micropellets, in agreement with a recent study published by Jones et al. [9]. These authors, studying the expression profiles of 157 human miRNA, identified 17 differentially expressed miRNAs in human OA in comparison to normal cartilage and they determined their relevance to chondrocyte function. In this sense, they postulated that miR-149 was downregulated in OA cartilage, this result is in agreement with our miRNA microarray analysis regarding miR-149, which was also downregulated in OA chondrocyte micropellets.

In previous reports hsa-miR-140 was down regulated and hsa-miR-146 was up regulated in OA cartilage [9,12,15]. In our study, the expression levels of hsa-miR-140 and hsa-miR-146 in the microarray analyses showed no statistical significant differences when comparing healthy and OA samples. However, hsa-miR-140 showed a tendency to be down regulated in OA and hsa-miR-146 showed a tendency to be up regulated in this pathology. Such discrepancies found among our results and those published on the literature could be due to the use of different microarray technologies, or to the use of cultured cell instead of tissue samples, or to the use of OA samples obtained from the different zones of the cartilage.

It is noteworthy that some of the miRNAs differentially expressed in chondrocyte that we identified in our study are novel as compared with those identified and published in the literature, e.g. hsa-miR-576-5p, hsa-miR-582-3p, hsa-miR-634, hsa-miR-641, hsa-miR-1227, suggesting that they may therefore represent new targets in articular cartilage.

The key molecular pathways potentially altered by the miRNAs differentially expressed in normal and OA chondrocyte micropellets, as predicted by the DIANA-mirPath web-based computational tool, include TGF-beta, Wnt, MAPK and mTOR signalling, focal adhesion and regulation of actin cytoskeleton among others. These results should be considered since Wnt signalling pathway has a role in OA pathology [51,52]. In particular these pathways are key inducers and regulators of joint development, and are involved in formation of bone, cartilage and also synovium [53]. For these reasons Wnt-family of proteins and signalling pathways are attractive targets for OA therapy. In this sense, products of the Wnt, frizzled, secreted frizzled-related protein (sFRP), Dickkopf and LDL-receptor-related protein gene families have crucial roles in the development and maintenance of bone, cartilage and joints [54]. In this sense, genes of the Wnt pathway are upregulated in the OA bone, suggesting their involvement not only in cartilage distortion but also in subchondral bone changes [55]. On the other hand, TGF-beta 1 induces cartilage extracellular matrix synthesis and tissue inhibitor of metalloproteinases-3 (TIMP-3), an important natural inhibitor of matrix metalloproteinases, aggrecanasses and TNF-alpha-converting enzyme, which are implicated in cartilage degradation and joint inflammation [56]. Moreover, genes belonging to the TGF-beta signalling pathway, which are supposedly targeted by the miRNAs differentially expressed in our work, regulate the chondrocyte differentiation and potentially the OA development [57]. Also, TGF-beta pathway regulates the expression of the superficial zone protein (SZP) in the superficial zone chondrocytes, protein implicated in the lubrication of the articular cartilage surface [58].

## Conclusions

Our potential miRNAtarget predictions and the signalling cascades (previously published in the literature to be involved in development, maintenance degradation of cartilage) altered by the differentially expressed miRNAs supports the potential involvement of the detected miRNAs in OA pathology. Taken together, we identified 7 miRNAs differentially expressed in normal and OA chondrocyte micropellets whose expression profiling may provide a useful clue for the pathophysiology research of OA. Due to the importance of miRNA in mediating the translation of target mRNA into protein, the identification of miRNA differentially expressed in normal and OA chondrocyte micropellets we report in this work could have important diagnostic and therapeutic potential. The study of miRNAs may lead to finding novel methods to diagnosis, treat and prevent OA. Further studies are needed to know the function of these miRNAs including the search of their target mRNA genes, which could lead to the development of novel therapeutic strategies for the OA treatment.

## Competing interests

The authors declare that they have no competing interests.

## Authors' contributions

SDP performed the molecular biology techniques, statistical and bioinformatic analyses. She drafted the manuscript. CC carried out molecular biology techniques and was involved in the conception and design of the study. EML carried out the cell culture experiments. THG carried out the histological analyses. NO and CFL collected and checked the samples. FJB conceived, designed, and coordinated the study and helped to draft the manuscript. All authors read and approved the final manuscript.

## Pre-publication history

The pre-publication history for this paper can be accessed here:

http://www.biomedcentral.com/1471-2474/13/144/prepub

## Supplementary Material

Additional file 1 **Stainings performed to determine the Mankin score in the normal and healthy cartilage samples used in the manuscript.** We only show 1 representative sample of healthy cartilage and 1 representative sample of OA cartilage. Click here for file
